# Letter to Editor on “Global disparities in healthcare resources and cancer burden a population-based systematic analysis of 171 Countries in 2022”

**DOI:** 10.1097/JS9.0000000000004777

**Published:** 2026-01-13

**Authors:** Yu Fu, Weiying Lu, Bangbei Wan

**Affiliations:** a Reproductive Medical Center, Hainan Women and Children’s Medical Center, Haikou, China; b Department of Urology, Haikou Affiliated Hospital of Central South University Xiangya School of Medicine, Haikou, China

*Dear Editor,*

Zhu *et al* present an important global analysis linking healthcare resource capacity with cancer incidence, mortality, and survival across 171 countries using GLOBOCAN 2022 data and WHO indicators of universal health coverage (UHC) and health expenditure^[1]^. Their findings align with established evidence that global cancer burden is profoundly shaped by socioeconomic development, health system capacity, and access to early detection and treatment^[2–4]^.

Three aspects of the study merit particular attention. First, the authors show that cancer incidence and estimated 5-year survival (1 − M/I) are substantially higher in countries with stronger UHC and higher current health expenditure (%GDP), whereas age-standardized mortality rates (ASMR) show little change. This mirrors well-documented epidemiologic transition patterns in which high-income countries diagnose more early-stage cancers and achieve markedly better survival despite higher incidence^[2]^. Second, the article distinguishes cancer-specific and regional disparities. Colorectal and breast cancers – common in high-income settings – demonstrate higher incidence but also improved survival due to widespread screening and established treatment protocols^[3]^. Conversely, cervical and liver cancers disproportionately affect low-resource regions, reflecting gaps in HPV vaccination, screening, antiviral therapy, and access to surgery and radiotherapy^[1]^. These findings align with global health estimates showing that most cancer deaths in low-income countries result from preventable or treatable malignancies^[4]^.

Third, Zhu *et al* use counterfactual modeling to estimate lives saved through improved health-system performance, projecting that >30% of global cancer deaths could be avoided if countries reached the survival levels of the best-performing systems^[1]^. This echoes recent Lancet Oncology and Cancer Atlas analyses demonstrating that scaling up essential cancer services – including pathology, surgical oncology, radiotherapy, and systemic therapy – could yield major survival gains, particularly in transitioning economies^[4,5]^.

Some limitations must be acknowledged. The ecological and cross-sectional nature of the analysis limits causal inference. Survival is approximated using M/I rather than patient-level data, which is vulnerable to registry completeness and diagnostic bias. Country-level analyses also mask profound within-country disparities documented across socioeconomic groups and regions^[6]^. Nonetheless, given the scarcity of harmonized global cancer data, the authors’ approach represents a methodologically sound and policy-relevant synthesis.

For surgical oncology communities, this work reinforces that inequities in UHC and health financing directly influence who develops cancer, who reaches surgical care, and who survives. Strengthening cancer services as part of UHC – particularly pathology infrastructure, timely surgical access, and affordable multimodal therapy – should be prioritized as nations advance along the development spectrum. The study by Zhu *et al* provides compelling quantitative evidence to support national cancer control strategies that emphasize early detection, equitable treatment access, and progressive expansion of health-system investment. This commentary was prepared in line with the TITAN 2025 guideline for transparent reporting^[7]^.

## Data Availability

No data was used in this Letter to the Editor.
